# Interplay of Eph-Ephrin Signalling and Cadherin Function in Cell Segregation and Boundary Formation

**DOI:** 10.3389/fcell.2021.784039

**Published:** 2021-11-05

**Authors:** David G. Wilkinson

**Affiliations:** The Francis Crick Institute, London, United Kingdom

**Keywords:** Eph receptor, ephrin, cadherin, cell segregation, boundary formation, hindbrain segmentation

## Abstract

The segregation of distinct cell populations to form sharp boundaries is crucial for stabilising tissue organisation, for example during hindbrain segmentation in craniofacial development. Two types of mechanisms have been found to underlie cell segregation: differential adhesion mediated by cadherins, and Eph receptor and ephrin signalling at the heterotypic interface which regulates cell adhesion, cortical tension and repulsion. An interplay occurs between these mechanisms since cadherins have been found to contribute to Eph-ephrin-mediated cell segregation. This may reflect that Eph receptor activation acts through multiple pathways to decrease cadherin-mediated adhesion which can drive cell segregation. However, Eph receptors mainly drive cell segregation through increased heterotypic tension or repulsion. Cadherins contribute to cell segregation by antagonising homotypic tension within each cell population. This suppression of homotypic tension increases the difference with heterotypic tension triggered by Eph receptor activation, and it is this differential tension that drives cell segregation and border sharpening.

## Introduction

The generation and maintenance of precisely patterned embryos requires that following the induction of specific cell or tissue types at the appropriate location, there are mechanisms to prevent intermingling between these distinct cell populations. In craniofacial development, this is exemplified by segmentation of the hindbrain to form a series of seven rhombomeres. The rhombomeres each have a distinct anteroposterior (A-P) identity which underlies the regional specification of neuronal cell types and branchial neural crest cells, and has a central role in coordinating the relationship between the central nervous system and craniofacial structures (Kiecker and Lumsden, 2005; Krumlauf and Wilkinson, 2021). At the molecular level, the segmentation and A-P patterning of the hindbrain involves the spatially-restricted expression of transcription factors, including Hox genes, mafB and Krox20, downstream of graded retinoic acid, Fgf and Wnt signals (Frank and Sela-Donenfeld, 2019; Krumlauf and Wilkinson, 2021). Initially, the borders of segmental gene expression are ragged (Irving et al., 1996; Cooke and Moens, 2002), reflecting imprecision in the formation and interpretation of graded signals (Zhang et al., 2012). Furthermore, the segmental pattern can potentially be scrambled by the intercalation of cells during tissue growth and convergent-extension movements (Fraser et al., 1990; Kimmel et al., 1994). Nevertheless, the initial fuzzy pattern of segmental gene expression is sharpened, at early stages through cell identity regulation (Addison et al., 2018), and later by mechanisms that drive cell segregation and prevent intermingling between segments (Fraser et al., 1990; Xu et al., 1995; Cooke et al., 2005; Kemp et al., 2009; Calzolari et al., 2014; Cayuso et al., 2019). Likewise, cell segregation mechanisms have critical roles throughout embryogenesis in the formation and maintenance of sharp borders between tissues and between regional domains within tissues (Dahmann et al., 2011).

**FIGURE 1:**
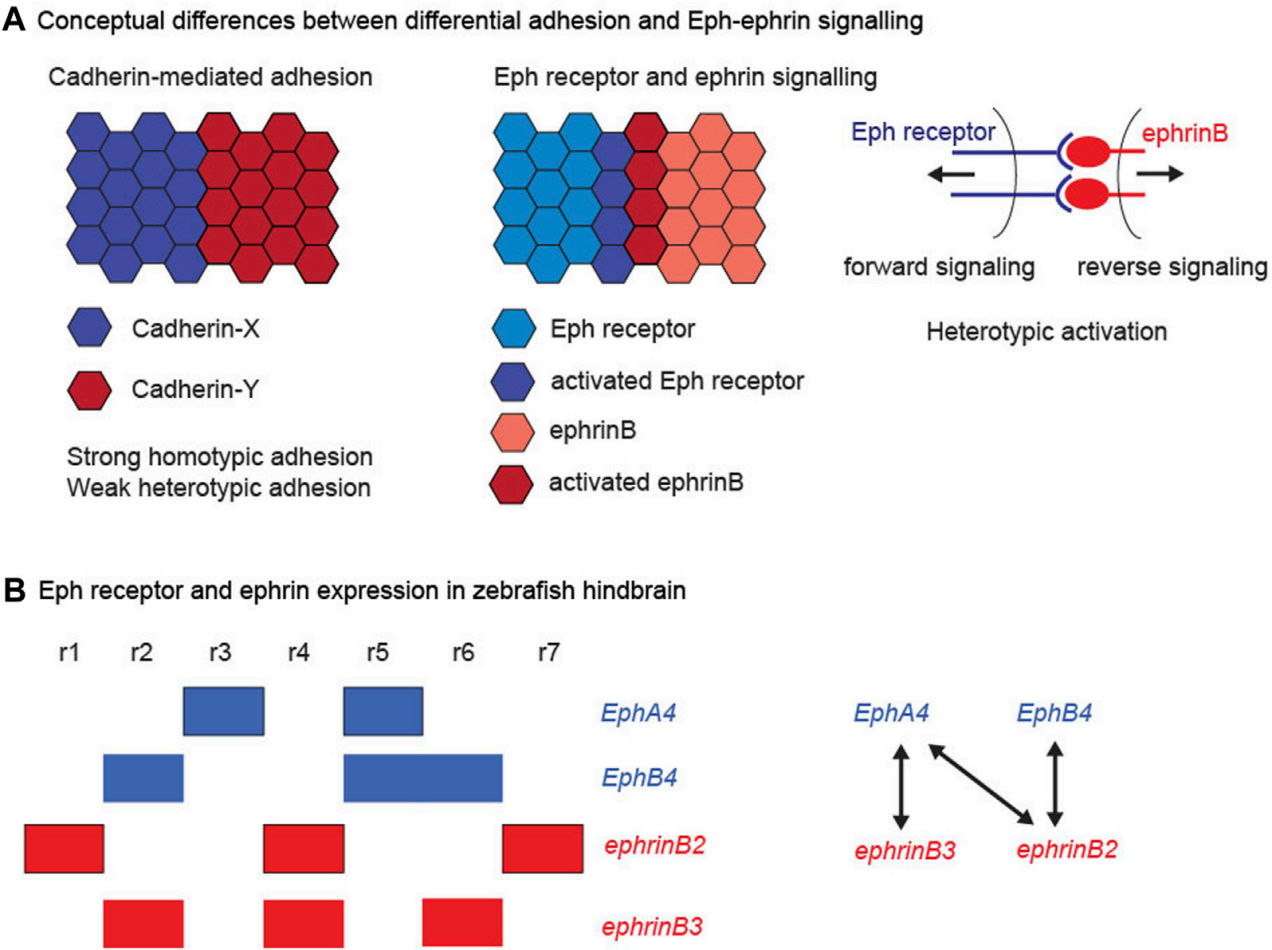
Cadherins, Eph receptors and ephrins. **(A)** Conceptual differences between differential adhesion and Eph-ephrin signalling. The left hand panel illustrates the differential adhesion hypothesis, in which the differential expression of cadherin family members leads to high homotypic adhesion and lower heterotypic adhesion. This global difference in adhesion properties can drive cell segregation. The right hand panels illustrate the complementary expression and bidirectional activation of an interacting Eph receptor and ephrin. This leads to Eph-ephrin activation at the heterotypic interface, which regulates cell responses that can drive cell segregation. **(B)** Eph receptor and ephrin expression in the zebrafish hindbrain. The left hand panel depicts the segmental expression of EphA4, EphB4, ephrinB2, and ephrinB3. The right hand panel depicts the high affinity interactions between the family members, which have complementary expression, thus leading to strong activation at the segment borders.

Two sets of molecular players have been identified that underlie distinct mechanisms of cell segregation. The first to be identified were the classical cadherins, transmembrane proteins that mediate strong homophilic adhesion and are linked to the intracellular cytoskeleton. The cadherins comprise a family of proteins, which are differentially expressed and in some cases associated with specific tissue types, for example E-cadherin in many epithelial tissues and N-cadherin in the neural epithelium and neural crest. Since homophilic adhesion is stronger than heterophilic adhesion, the expression of different cadherin family members in adjacent cell populations leads to differential adhesion, which *in vitro* assays showed can drive cell segregation (Foty and Steinberg, 2005; Steinberg, 2007; Steinberg and Takeichi, 1994). Cadherin-mediated differential adhesion has been found to act in cell segregation *in vivo*, for example for cadherin6 and R-cadherin in subdivisions of the developing forebrain (Inoue et al., 2001) and for MN-cadherin in motor neuron cell types (Price et al., 2002). The second mechanism involves signalling at the interface of cell populations mediated by Eph receptors and ephrins. Eph-ephrin signalling leads to cell responses at the heterotypic interface that can drive cell segregation, and underlies the formation and maintenance of boundaries in many tissues during vertebrate development (Batlle and Wilkinson, 2012; Cayuso et al., 2015; Fagotto, 2020; Fagotto et al., 2014; Klein, 2012). Thus cell segregation can be driven by global differences in cell-cell adhesion or by cell responses to signalling between 2 cell populations (Figure 1A). This raises the question of whether cadherin-mediated adhesion and Eph-ephrin signalling act as alternative mechanisms used at distinct sites, and/or work together in some situations. In support of the latter possibility, both Eph-ephrin signalling and cadherins are required for cell segregation in cell culture assays (Cortina et al., 2007; Taylor et al., 2017), in clustering of cells to form discrete sympathetic ganglia (Kasemeier-Kulesa et al., 2006) and in the sharpening of specific borders (Kesavan et al., 2020). This review will first summarise current understanding of the mechanisms by which Eph-ephrin signalling drives cell segregation, and then focus on the relationships and potential cross-talk between Eph-ephrin signalling and cadherins.

## Mechanisms of Eph-Ephrin Signalling in Cell Segregation

Eph receptors are a family of transmembrane receptor tyrosine kinases with an extracellular domain that interacts with ephrin ligands. Ephrins are also membrane-bound, either through a GPI linkage (ephrinAs) or through a transmembrane domain (ephrinBs), and with a few exceptions interact with the EphA and EphB subfamilies of receptors, respectively (Gale et al., 1996a; Gale et al., 1996b). Binding of Eph receptor to ephrin leads to clustering of the complex, and this triggers intracellular signal transduction downstream of both components, termed forward and reverse signalling, respectively (Pasquale, 2008; Klein, 2012). Both forward and reverse signalling involve tyrosine phosphorylation, either by the receptor tyrosine kinase domain or by cytoplasmic tyrosine kinases that phosphorylate the intracellular domain of ephrinBs. In addition, there are kinase-independent pathways, which include recruitment of PDZ domain proteins to the C-terminus of Eph and ephrinB proteins. Since both Eph receptors and ephrins are membrane-bound this leads to bidirectional cell contact-dependent signalling, but signalling may also occur at a distance through secretion of ligand-bearing exosomes (Gong et al., 2016).

Expression of high affinity Eph receptor and ephrin binding partners often occurs in complementary domains, such that interactions occur at boundaries of distinct tissues or subdivisions within tissues (Gale et al., 1996b). For example, in the zebrafish hindbrain, expression of EphB4 in r2, r5, and r6 is complementary to ephrinB2 in r1, r4, and r7, and expression of EphA4 in r3 and r5 is complementary to ephrinB3 in r2, r4, and r6, as well as to ephrinB2 (Figure 1B). There is extensive evidence that signalling through these Eph receptors and ephrins underlies the sharpening of segment borders in the hindbrain (Xu et al., 1995; Xu et al., 1999; Cooke et al., 2001; Cooke et al., 2005; Kemp et al., 2009; Sela-Donenfeld et al., 2009; Calzolari et al., 2014; Cayuso et al., 2019). Studies of cell segregation and border formation *in vivo* have found a dominant role of Eph receptor forward signalling through kinase-dependent pathways (Cayuso et al., 2019; O'Neill et al., 2016; Rohani et al., 2014), although reverse signalling through ephrinBs could also contribute (Wu et al., 2019). Eph receptor activation can lead to three types of cell response that can drive cell segregation and restrict intermingling across borders: (1) A decrease in cadherin-mediated adhesion, which will lead to lower heterotypic compared with homotypic adhesion. (2) An increase in cortical tension by contraction of cortical actomyosin, through Rho kinase activation leading to myosin light chain phosphorylation and myosin II activation. (3) The repulsion and directional migration of cells following cell-cell contact by repolarising the front-rear orientation of migrating cells. Which of these mechanisms is utilised may relate to whether the boundary is within an epithelial tissue, at the interface of tissues, or between mesenchymal cells. For example, the generation of sustained tension requires adhesive contacts between cells (Maitre et al., 2012), as occurs within epithelial tissues, whereas migratory mesenchymal cells can repel and move away from each other after contact.

## Requirement for Cadherins in Eph-Ephrin-Mediated Cell Segregation

One situation in which Eph-ephrin signalling and cadherins are both required for cell segregation is illustrated by studies of the formation of sympathetic ganglia. Initially the ganglionic cells are spread out along the A-P axis, and then migrate to form a series of discrete clusters (Kasemeier-Kulesa et al., 2006). This segregation is driven by Eph-mediated repulsion that excludes ganglionic cells from interganglionic regions (Krull et al., 1997; Wang and Anderson, 1997), together with N-cadherin-mediated adhesion between ganglionic cells (Kasemeier-Kulesa et al., 2006). Such segregation can be explained by synergy between parallel roles of Eph-ephrin-mediated repulsion and cadherin-mediated adhesion (Kasemeier-Kulesa et al., 2006), in which cells are responding to a pre-pattern of ephrin expression in the adjacent mesenchyme. This is a different scenario from cell segregation between distinct tissues, or subdivisions within a tissue, in which cells are not responding to a prepattern in another tissue. Nevertheless, as discussed below, a similar synergy of repulsion and adhesion mechanisms may contribute to cell segregation.

Studies in cell culture assays and *in vivo* have suggested that cadherins are required for cell segregation that is driven by Eph-ephrin signalling. For example, in cell culture assays, EphB2 and ephrinB1-expressing colorectal cancer cell lines were found to segregate from each other, but this segregation was disrupted by knockdown of E-cadherin (Cortina et al., 2007). Likewise, EphB2 and ephrinB1-expressing HEK293 cells segregate from each other (Poliakov et al., 2008), and this is decreased by knockdown of N-cadherin (Taylor et al., 2017). Assays in which cells from different rhombomeres from chick embryos are mixed *in vitro* suggested a requirement for cadherins in segregation (Wizenmann and Lumsden, 1997), which subsequent work found is driven by Eph receptor and ephrin signalling (Xu et al., 1999; Cooke et al., 2005; Kemp et al., 2009; Sela-Donenfeld et al., 2009; Calzolari et al., 2014; Cayuso et al., 2019). Similarly, recent work has found that both Eph-ephrin signalling and N-cadherin function are required for sharpening of the mid-hindbrain boundary (Kesavan et al., 2020). At the boundaries in the midbrain and hindbrain, and in the cell culture assays, there is no apparent difference in expression of the relevant cadherin between the segregating cell populations. It is therefore unlikely that the segregation involves synergy between differential adhesion and Eph-ephrin-mediated repulsion. There are a number of other potential explanations for the involvement of cadherins: (1) Cadherins are required for the correct expression and/or activation of Eph receptors and ephrins; (2) Eph receptors drive cell segregation through the modulation of cadherin function; (3) Cell segregation involves a balance in cell responses in which Eph receptor signalling and cadherins oppose each other. Evidence in support of these potential relationships are discussed below.

## Regulation of Eph Receptor Expression and Localisation by Cadherins

The switching of cadherin expression from E-cadherin to N-cadherin is an important factor in the epithelial to mesenchymal transition and migratory behaviour of neural crest cells (Scarpa et al., 2015). Interestingly, in ES cells mutant for E-cadherin there is a decrease in EphA2 expression and increased expression of EphB4, ephrinA1, ephrinA2, ephrinB1, ephrinB2, and ephrinB3 (Orsulic and Kemler, 2000). The decrease in EphA2 expression was rescued by transfection of E-cadherin but not N-cadherin, and significantly the overexpression of E-cadherin in NIH3T3 cells led to an increase in EphA2 expression (Orsulic and Kemler, 2000). Furthermore, E-cadherin was found to be required for the localisation and phosphorylation of EphA2 at cell-cell contacts (Zantek et al., 1999; Orsulic and Kemler, 2000). It is therefore important to consider the possibility that knockout or knockdown of cadherin genes have led to a change in the developmental expression of Eph receptors and ephrins, and/or a decrease in cell surface localisation and activation of Eph receptors.

Interestingly, there is also evidence for regulatory relationships in the converse direction, in which Eph-ephrin activation can increase the level of cadherins at the cell surface. The segregation and migration of Schwann cells during peripheral nerve regeneration is mediated by EphB2 activation by ephrinB ligands, which through Sox2 leads to relocalization of N-cadherin to intercellular contacts of Schwann cells (Parrinello et al., 2010). Similarly, Eph-ephrin signalling in the pharyngeal endoderm leads to an increase in junctional E-cadherin, in this case through a pathway that involves targeting of Pak2a to the plasma membrane and Wnt4/Cdc42 activation (Choe and Crump, 2015). Another pathway has been found in neural crest cells, in which ephrinB2 negatively regulates E-cadherin recycling, and upon activation by EphB receptor this inhibition is relieved, leading to increased E-cadherin at the cell surface (Yoon et al., 2018). Intriguingly, recent work has found a direct interaction between E-cadherin and ephrinB1 (Shafraz et al., 2020), suggesting another way in which they could influence the subcellular localisation of each other.

## Modulation of Cadherin Function by Eph Receptors

**FIGURE 2:**
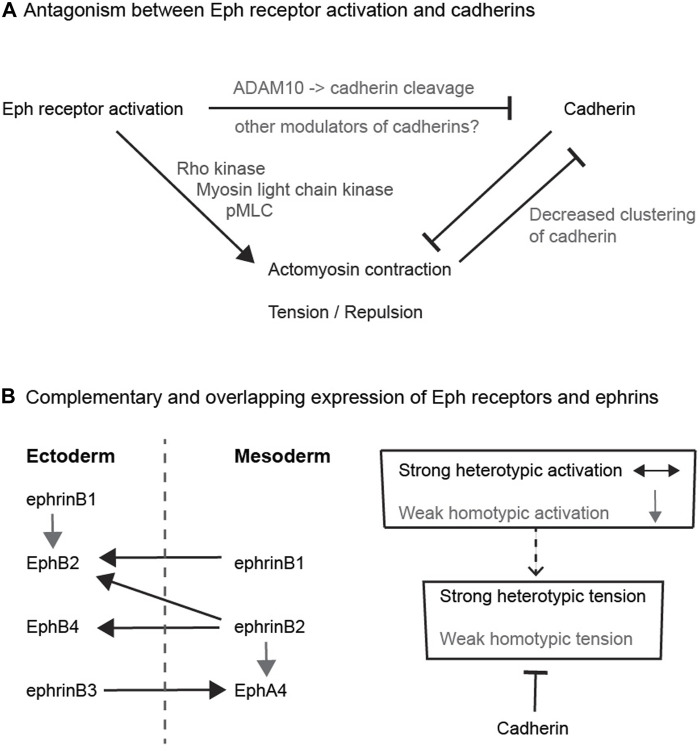
Potential relationships between Eph receptors and cadherins **(A)** Antagonism between Eph receptor activation and cadherins. The diagram depicts the antagonism between adhesion and cortical tension, and pathways that have been found to link Eph receptor activation to decreased cadherin function. **(B)** Complementary and overlapping expression of Eph receptors and ephrins. The left hand diagram depicts the expression pattern and interactions of Eph receptors and ephrins in ectoderm and mesoderm in *Xenopus*. Each tissue expresses a combination of Eph receptors and ephrins that leads to strong heterotypic activation (black arrows) of Eph receptors in both directions that underlies increased cortical tension and cell repulsion. In addition, there is weaker homotypic activation of Eph receptor by low-level or low-affinity ephrin co-expressed within each tissue (grey arrows). The right hand diagram depicts that cadherins may serve to suppress the weaker homotypic tension due to overlapping Eph-ephrin expression, whereas strong heterotypic Eph receptor activation dominates over cadherin-mediated adhesion.

Several pathways have been uncovered that link Eph-ephrin interactions to a decrease in cadherin function (Figure 2A), such that there is less adhesion across the heterotypic interface than for homotypic contacts. In principle, this quantitative difference between homotypic and heterotypic adhesion could drive cell segregation (Steinberg, 2007). In one mechanism, the extracellular domain of EphB receptors interacts with the metalloproteinase, ADAM10, which is activated upon binding to ephrinB1 (Solanas et al., 2011). Activated ADAM10 cleaves the extracellular domain of E-cadherin, leading to shedding of cadherin and decreased adhesion at the interface (Solanas et al., 2011). Furthermore, blocking of ADAM10 disrupts Eph receptor-mediated cell segregation in cell culture assays and in the intestinal epithelium (Solanas et al., 2011). A related role has been found in the cochlear sensory epithelium, in which EphA4-ephrinB2 interactions lead to E-cadherin cleavage by ADAM10 that enables separation of adjacent cells (Defourny et al., 2019). In addition to cleaving E-cadherin, ADAM10 mediates cleavage of ephrinA ligands from the cell surface following interaction with EphA receptors (Hattori et al., 2000; Janes et al., 2005; Atapattu et al., 2012). Since Eph-ephrin interactions can potentially mediate strong adhesion, such proteolytic cleavage, and/or endocytosis of Eph receptors and ephrins (Marston et al., 2003; Zimmer et al., 2003), are essential for cells to disengage following their interaction.

In a second type of mechanism, EphB4 activation at the notochord-presomitic mesoderm boundary was shown to decrease the clustering of C-cadherin compared with homotypic contacts (Fagotto et al., 2013). The decrease in cadherin clustering was regulated by myosin activation leading to increased cortical tension (Fagotto et al., 2013). Since clustering of cadherins increases adhesion, this pathway leads to a decrease in adhesion at the heterotypic interface. Finally, proteomic studies to identify tyrosine phosphorylation targets downstream of Eph receptor and ephrin activation have found other pathways that potentially modulate cadherin function. For example, analysis of EphB2 and ephrinB1 expressing HEK293 cells found a decrease in tyrosine phosphorylation of several mediators of adhesion, and of regulators of cadherin endocytosis and stability, including Cadm1, Pcdh7, Ctnnd1, Cttn, and Dcs2 (Jorgensen et al., 2009). Furthermore, forward and reverse signalling was found to have distinct effects on the different multiple tyrosine phosphorylation sites of Cttn and Ctnnd1. Ctnnd1 (p120-catenin) is especially interesting as it both stabilises E-cadherin at the cell surface and suppresses RhoA function, and thus a decrease in its activity leads to less adhesion and greater tension (Yu et al., 2016). It will be interesting to determine whether the changes in tyrosine phosphorylation downstream of Eph-ephrin signalling modulate the function of these proteins in the regulation of adhesion.

These findings are consistent with the idea that cadherins are required for Eph-ephrin-mediated cell segregation because decreased heterotypic adhesion is driving segregation. This raises the question of the relative contribution of decreased adhesion compared with increased cortical tension or cell repulsion responses to Eph receptor activation, that can also potentially drive cell segregation. This was addressed in studies in which cell responses were quantitated and the measurements used in computer simulations of cell segregation and border sharpening. The formation of a sharp border between ectoderm and mesoderm in *Xenopus* depends upon activation of Eph receptors that leads to high heterotypic tension and cell repulsion at the interface (Rohani et al., 2011; Canty et al., 2017). In computer simulations it was found that cell segregation and border sharpening is efficiently driven when heterotypic tension is higher than the homotypic tension that occurs within each cell population (Canty et al., 2017). In contrast, global differences in tension or adhesion between the 2 cell populations are less efficient (Canty et al., 2017). This prediction was tested in cell segregation assays and it was found that, unlike Eph-ephrin signalling, differential expression of E-cadherin and N-cadherin does not drive the separation of tissues (Canty et al., 2017).

A similar conclusion came from use of cell culture assays with EphB2 and ephrinB1 expressing HEK293 cells (Taylor et al., 2017). Analysis of cell behaviour at low density revealed strong repulsion and transient adhesion following heterotypic contacts between cells (Taylor et al., 2017). Cell repulsion involves a rapid collapse of cell processes at the site of heterotypic contact, and reorientation of front-rear polarity so that the cells migrate away from each other. This is likely mediated by localised activation of Rho family GTPases that are targets of Eph receptor signalling and regulate the actin cytoskeleton (Pasquale, 2008; Klein, 2012). It may also involve Par proteins that regulate the front-rear polarity of migrating cells since these are among the phosphorylation targets of activated EphB2 (Jorgensen et al., 2009). Since HEK293 cells are intrinsically motile at low density, cell repulsion need not lead to an increase in overall migration, but rather a reorientation after each contact. Agent-based simulations with the measured values of repulsion and cell contact duration found that heterotypic repulsion drives efficient segregation and border sharpening, whereas a heterotypic decrease in adhesion alone does not (Taylor et al., 2017). Taken together, these studies suggest that increased heterotypic repulsion or tension drive efficient segregation and are the principal mechanisms by which Eph-ephrin signalling leads to border sharpening. Consistent with this, a number of studies have revealed increased tension and cell repulsion at sites of Eph-ephrin interactions at borders of tissues and in cell segregation assays (Fagotto et al., 2013; Kindberg et al., 2021; O'Neill et al., 2016; Rohani et al., 2011). Nevertheless, it remains possible that decreased cadherin-mediated adhesion at heterotypic contacts drives Eph-ephrin-mediated cell segregation in some contexts. Another possibility is that the decrease in cadherin-mediated adhesion can promote cell segregation because there is antagonism between tension and adhesion, as discussed below. A decrease in heterotypic adhesion may thus contribute to the increase in tension and triggering of repulsion following Eph receptor activation.

## Antagonism Between Adhesion and Tension

There is an intimate mechanistic relationship in which cadherin-mediated adhesion antagonises the generation of tension and cell repulsion by actomyosin contraction in the cell cortex (Winklbauer, 2015). Conceptually, this is illustrated by the effect of increasing the strength of adhesion between two cells, which increases their area of contact, and is decreased by actomyosin contraction that generates tension (Lecuit and Lenne, 2007). Consideration of the forces that underlie cell segregation suggest that the binding energy of cadherins alone makes a minor contribution, and rather cadherins mainly act by decreasing cortical tension (Winklbauer, 2015). Cadherin-mediated adhesion and actomyosin contraction downstream of Eph-ephrin signalling may thus act in opposition to regulate the strength of cortical tension.

At first sight, it is not intuitive that such antagonism between cadherins and Eph-ephrin signalling would contribute to cell segregation. However, a potential role is suggested by detailed studies of Eph receptor and ephrin expression. Initial studies emphasised that interacting Eph receptors and ephrins have complementary expression, such that interactions only occur at the interface (Gale et al., 1996b). However, more comprehensive analyses have found that there are also some overlaps in expression, which have been best described for ectoderm and mesoderm in *Xenopus*, where Eph receptor activation has a critical role in preventing cell intermingling (Rohani et al., 2011; Rohani et al., 2014). It was found that each cell population expresses a cocktail of Eph receptors and ephrins: EphB4, EphB2, ephrinB3 and ephrinB1 in ectoderm, and EphA4, ephrinB2 and ephrinB1 in mesoderm (Rohani et al., 2014); (Figure 2B). The activation of Eph receptors following homotypic or heterotypic cell contacts can be predicted from measurements of Eph-ephrin binding affinity: EphB4 has high affinity only for ephrinB2, EphA4 binds to ephrinB3 and ephrinB2, and EphB2 binds to ephrinB1 and ephrinB2 (Gale et al., 1996a; Gale et al., 1996b). Consequently, there is heterotypic activation of EphB4 by ephrinB2, of EphB2 by ephrinB2 and ephrinB1, and of EphA4 by ephrinB3, such that forward signalling occurs in both directions at the interface of ectoderm and mesoderm (Rohani et al., 2011; Rohani et al., 2014). In addition, there is homotypic activation of EphA4 by ephrinB2, and of EphB2 by ephrinB1, within ectoderm and mesoderm. This predicts that in addition to strong Eph receptor activation at the tissue interface, there is Eph receptor activation within each cell population (Figure 2B). Furthermore, depletion of cadherins leads to an increase in homotypic tension and repulsion of cells, consistent with antagonism between adhesion and repulsion (Rohani et al., 2014). The role of such antagonism was tested by analysing the effect of overexpressing cadherin in ephrin-expressing cells juxtaposed with Eph receptor-expressing cells (Canty et al., 2017). It was found that cadherin expression increased cohesion and decreased tension at homotypic contacts, leading to a greater difference between heterotypic and homotypic tension that drives cell segregation (Canty et al., 2017). Importantly, this role of cadherins in suppressing homotypic tension has a much stronger input into cell segregation than differential cadherin-mediated adhesion (Canty et al., 2017).

Similar insights have come from cell culture assays with HEK293 cell lines over-expressing EphB2 or ephrinB1 (Taylor et al., 2017). The segregation and formation of sharp borders between EphB2 and ephrinB1 expressing cells is driven by strong heterotypic activation leading to cell repulsion at low density (Taylor et al., 2017) and increased cortical tension when cells are confluent (Kindberg et al., 2021). There is also a repulsion response, that is less strong. following homotypic interactions between EphB2 expressing cells, which is due to overlapping expression of ephrinB family members intrinsic to HEK293 cells (Taylor et al., 2017). Cell segregation and formation of a sharp border are decreased by knockdown of N-cadherin, the main cadherin expressed by these cells. Measurements of cell behaviour found that N-cadherin knockdown leads to a decrease in adhesive interactions and increase in cell repulsion. The increase in cell repulsion was much greater for homotypic interactions than for heterotypic interactions, leading to a narrowing of the quantitative difference between heterotypic and homotypic repulsion (Taylor et al., 2017). N-cadherin may thus counterbalance the low level cell repulsion response to weak homotypic Eph receptor activation, whereas strong heterotypic Eph receptor activation leads to repulsion that is little affected by N-cadherin. Since the difference between heterotypic versus homotypic cell responses drives segregation, cadherins facilitate segregation by suppressing homotypic tension and repulsion (Canty et al., 2017; Taylor et al., 2017).

A recent study has presented a different perspective on the role of cadherins and EphB2 in cell segregation (Kindberg et al., 2021). Using cell cultures at high density, in which cells are in sustained contact and migration is constrained, the segregation of EphB2 and ephrinB1 cells was shown to be driven by greater heterotypic than homotypic cortical tension. This high heterotypic tension is dependent on actomyosin contraction downstream of Rho kinase (ROCK) and myosin light chain kinase (MLCK). It was found that blocking of cadherin-mediated adhesion by depletion of calcium had no significant effect on EphB2/ephrinB1 cell segregation. This result contrasts with other work with the same cell lines that found a decrease in cell segregation and border sharpening following knockdown of N-cadherin (Taylor et al., 2017). The apparent discrepancy may reflect differences in the sensitivity of the methods used to quantitate segregation, and/or in the effects of calcium depletion compared with N-cadherin knockdown. Interestingly, both studies found that following mixing with ephrinB1 cells, EphB2 cells aggregate into clusters in which there is increased homotypic contact between cells (Taylor et al., 2017; Kindberg et al., 2021). Kindberg et al. (2021) present evidence that this homotypic EphB2 cell-cell interaction does not involve cadherins, but rather is driven by a high level of cortical tension at the cell-medium interface. In contrast, Taylor et al. (2017) suggest a role of N-cadherin since its knockdown leads to an increase in repulsion and decrease in the duration of contact following homotypic interaction of EphB2 cells. A potential complication to experiments with these cell lines is that heterotypic interactions lead to a major decrease in the steady state level of EphB2 due to endocytosis of receptor-ligand complexes (Wu et al., 2019). Furthermore, HEK293 cells endogenously express low levels of ephrinB ligands that impact on EphB2 cell segregation and likely underlie homotypic repulsion (Taylor et al., 2017). The decrease in homotypic EphB2 cell repulsion and increase in cell contact that occurs following heterotypic activation of EphB2 by ephrinB1-expressing cells (Taylor et al., 2017) may thus be due to the decrease in the steady state level of EphB2. It will be interesting to determine whether this effect of heterotypic Eph receptor activation on homotypic cell interactions in a cell culture model is relevant to cell segregation *in vivo*.

## Summary and Perspectives on Border Sharpening in the Hindbrain

Two main themes have emerged from studies of the relationships between Eph-ephrin signalling and cadherins. The first is that Eph receptors can act through multiple pathways to inhibit cadherin function (Figure 2A). However, although this can lead to a heterotypic decrease in adhesion, it is increased cortical tension or repulsion downstream of Eph receptor activation that is the principal driver of cell segregation. Since cadherins antagonise cortical tension, the inhibition of cadherin function may thus contribute to the increase in tension at the heterotypic interface. The second theme is that this antagonism by cadherins may suppress homotypic tension within each cell population. This tension can be due to overlapping expression of Eph receptors and ephrins that leads to weak activation at homotypic contacts, as shown for the ectoderm-mesoderm border (Figure 2B). Another possibility is suggested by evidence for ligand-independent responses of cells to Eph receptors and ephrins (Noren et al., 2009; Daar, 2012; Miao and Wang, 2012; Lisabeth et al., 2013). Such ligand-independent pathways can regulate actomyosin contraction and cell morphology (Bochenek et al., 2010; Cayuso et al., 2016), and may thus increase tension at homotypic contacts. Whether through overlapping Eph-ephrin expression, or ligand-independent pathways, an increase in homotypic tension is counteracted by cadherin-mediated adhesion, thus increasing the difference between heterotypic and homotypic tension that drives cell segregation and border sharpening.

A model for how cells segregate to form sharp segment borders in the hindbrain can be proposed based on analyses of Eph-ephrin function in other tissues, summarised above, together with studies in the hindbrain itself. The initial ragged border of hindbrain segments is transformed into a sharp and straight border through a combination of cell identity regulation (Zhang et al., 2012; Addison et al., 2018) and Eph-ephrin-mediated cell segregation (Xu et al., 1995; Xu et al., 1999; Cooke et al., 2001; Cooke et al., 2005; Kemp et al., 2009; Calzolari et al., 2014; Cayuso et al., 2019). Cell segregation is likely driven by increased cortical tension at the heterotypic interface, principally through Eph receptor forward signaling (Cayuso et al., 2019) that acts through ROCK and MLCK to increase actomyosin contraction. An actomyosin cable is detected at the segment borders after they have sharpened, which generates sustained tension required to maintain border sharpness (Calzolari et al., 2014). In addition, through the Yap/Taz pathway the sustained tension induces formation of specialised hindbrain boundary cells (Cayuso et al., 2019) and regulates the balance of cell proliferation and neurogenesis (Voltes et al., 2019).

In this model, the complementary expression of interacting Eph receptors and ephrins (Figure 1B) leads to heterotypic activation at the segment borders which drives cell segregation. However, this does not account for the finding that mosaic knockdown of EphA4 (expressed in r3 + r5) leads to segregation of the knockdown cells within r3 + r5 to the segment borders (Cooke et al., 2005). Similarly, mosaic knockdown of ephrinB2 leads to cell segregation within the ephrinB2-expressing segments (Kemp et al., 2009). These findings can be explained if there is weak activation of Eph receptors within segments due to overlapping expression with ligands, analogous to findings for the ectoderm-mesoderm border (Rohani et al., 2014), and/or ligand-independent pathways that increase cortical tension. Such homotypic tension is counter-balanced by N-cadherin-mediated adhesion, thus increasing the difference between homotypic and heterotypic tension. This predicts that hindbrain border sharpening is disrupted in a zebrafish N-cadherin mutant (parachute), similar to findings for the midbrain-hindbrain border (Kesavan et al., 2020). Mosaic knockdown of Eph receptors or ephrins will consequently create a decrease in cortical tension compared with cells lacking the knockdown, thus leading to cell segregation within the segments. A comprehensive understanding of Eph receptor and ephrin expression and function in the hindbrain will be needed to test these ideas.

More broadly, in the context of craniofacial development, it will be interesting to explore potential relationships between Eph-ephrin signalling and cadherins in branchial neural crest migration. Early studies found complementary expression between EphA4 plus EphB1 and ephrinB2 in branchial neural crest streams in *Xenopus*, and that dominant negative blocking and ephrin overexpression led to incorrect migration into branchial arches (Smith et al., 1997). Intriguingly, N-cadherin has an important role in branchial neural crest cell migration in which it promotes homotypic contact repulsion (contact inhibition of locomotion) by polarising Rac activity to be stronger distal to the cell-cell contact (Theveneau et al., 2010; Scarpa et al., 2015). This suggests a distinct relationship, in which rather than suppressing repulsion responses to Eph receptor activation, N-cadherin itself promotes repulsion. It will therefore be important to understand the mechanistic basis of context-dependent functions of N-cadherin in cell repulsion and adhesion and how these affect the interplay with Eph-ephrin signalling.
